# P-75 - PRELIMINARY DESCRIPTION OF RECENT EPIDEMIOLOGY AND CLUSTERING OF STREPTOCOCCUS PNEUMONIAE SEROTYPE 4 INVASIVE PNEUMOCOCCAL DISEASE IN SCOTLAND, 2016–2025

**DOI:** 10.1093/pubmed/fdag046.094

**Published:** 2026-07-09

**Authors:** Mr James Munro, Ms Laura MacDonald, Dr Cheryl L Gibbons, Dr Taimoor Hasan, Dr Claire Cameron

**Affiliations:** Public Health Scotland; Public Health Scotland; Public Health Scotland; Public Health Scotland; Public Health Scotland

## Abstract

**Background:**

Streptococcus pneumoniae serotype 4 Invasive Pneumococcal Disease (IPD) has re-emerged globally since 2020. A vaccine-preventable serotype mainly seen in adults, it has been linked to uneven vaccination coverage and social vulnerability, particularly in Europe and North America.

**Objectives:**

To examine recent trends of Streptococcus pneumoniae serotype 4 IPD in Scotland, including incidence, demographics, geography and molecular clustering.

**Methods:**

All lab-confirmed serotype 4 IPD cases from 2016–2025 in Electronic Communication of Surveillance Scotland (ECOSS) were reviewed, summarising demographics and geography. Incidences between periods: 2016–2022 and 2023–2025 were compared using IRR, SIR and Fisher’s exact tests (for sparse data), examining changes in Health Board area contributions. MLST clustering was analysed with chi-square, standardised residuals and paired Fisher’s exact tests.

**Results:**

Forty-five Scotland-resident cases were identified. Annual numbers rose from 2023 (8 cases) through 2024 (17 cases) to November 2025 (12 cases) following several years of lower activity, including the COVID-19 pandemic period. Mean age was 54.42 (median 54.00; range 20-85; interquartile range 43 to 69). 64.44% (n=29) were male. 48.89% of cases were in the NHS Grampian area. The remainder occurred in eight other Health Boards. National incidence in 2023–2025 was significantly higher than in 2016–2022 (IRR 10.54; 95% CI 4.91–22.64). Grampian showed significantly elevated incidence of serotype 4 in 2023–2025 (SIR 3.81; 95% CI 2.33–5.88). There was no significant increase in Grampian’s share between periods (p = 0.2427). MLST results indicated ST15063 predominated in 2023-2025, clustered in Grampian.

**Conclusions:**

Serotype 4 IPD increased in Scotland in 2023-2025. Epidemiological and molecular findings indicate geographic lineage clustering although the significance of this is unclear. Continued molecular/genomic surveillance is required to assess risk and guide appropriate public health responses. Further investigations, including linkage to demographic data, may help indicate which groups are at greater risk of serotype 4 IPD in Scotland.

